# Preclinical Models for Studying Fuchs Endothelial Corneal Dystrophy

**DOI:** 10.3390/cells14070505

**Published:** 2025-03-28

**Authors:** Fancheng Sun, Lexie W. Q. Xi, Wesley Luu, Myagmartsend Enkhbat, Dawn Neo, Jodhbir S. Mehta, Gary S. L. Peh, Evelyn K. F. Yim

**Affiliations:** 1 Department of Chemical Engineering, University of Waterloo, Waterloo, ON NL2 3G1, Canada; f26sun@uwaterloo.ca (F.S.); lexie.xi@uwaterloo.ca (L.W.Q.X.); wesley.luu@uwaterloo.ca (W.L.); menkhbat@uwaterloo.ca (M.E.); 2 Singapore Eye Research Institute, Singapore 169856, Singapore; dawn.neo.j.h@seri.com.sg (D.N.); jodhbir.s.mehta@singhealth.com.sg (J.S.M.); 3 Ophthalmology Academic Clinical Program, Duke-NUS Graduate Medical School, Singapore 169857, Singapore; 4 Centre for Biotechnology and Bioengineering, University of Waterloo, Waterloo, ON NL2 3G1, Canada; 5 Waterloo Institute for Nanotechnology, University of Waterloo, Waterloo, ON NL2 3G1, Canada

**Keywords:** Fuchs Endothelial Corneal Dystrophy (FECD), pathogenesis, corneal endothelial cells (CEnCs), disease modeling

## Abstract

Fuchs Endothelial Corneal Dystrophy (FECD) is a corneal endothelial disease that causes microenvironment alterations and endothelial cell loss, which leads to vision impairment. It has a high global prevalence, especially in elderly populations. FECD is also one of the leading indications of corneal transplantation globally. Currently, there is no clearly defined canonical pathway for this disease, and it has been proposed that the combinatorial effects of genetic mutations and exogenous factors cause FECD. Clinical studies and observations have provided valuable knowledge and understanding of FECD, while preclinical studies are essential for gaining insights into disease progression and mechanisms for the development and testing of regenerative medicine therapies. In this review, we first introduce the proposed genetic and molecular pathologies of FECD. Notably, we discuss the impact of abnormal extracellular matrix deposition (guttata), endothelial-to-mesenchymal transition, cell senescence, and oxidative stress on the pathology and etiology of FECD. We review and summarize the in vitro cell models, ex vivo tissues, and in vivo animal models used to study FECD. The benefits and challenges of each model are also discussed.

## 1. Introduction

Fuchs endothelial corneal dystrophy (FECD) is a condition characterized by a dysfunctional corneal endothelium (CE) and the accumulation of posterior banded collagen, known as guttata, on the Descemet’s membrane due to abnormal extracellular matrix (ECM) deposition. CE plays a critical role in maintaining corneal deturgescence. When it fails, it can lead to stromal edema, resulting in decreased visual acuity, and subsequent corneal blindness can occur [1,2]. The prevalence of FECD varies among different regions, sexes, and ethnic groups [1,2,3,4,5,6]. It is one of the most common genetic disorders affecting CE [3,7,8]. In 2023 [9], over 19,900 corneal transplant surgeries were performed in the United States due to FECD-associated corneal edema, making it the primary indication for corneal transplantation (2023 Eye Banking Statistical Report, Eye Bank Association of America).

Despite the abundance of data confirming multiple pathologies leading to FECD, a unified theory remains elusive [10]. Several genetic mutations have been recognized to be associated with this disease. Notably, early onset FECD has been linked to point mutations in the collagen VIII α2-subunit chain (*COL8A2*) [11], while mutations in SLC4A11, responsible for the boron transporter pump [12,13,14,15,16,17,18], and trinucleotide repeat expansions in the transcription factor 4 (TCF4) region on chromosome 18 have also been implicated in late-onset FECD [4,12,18,19,20,21,22,23,24,25,26]. In addition, factors such as age, sex, and environment (ultraviolet light exposure) have been proposed as potential contributors to the development of FECD [2]. Given the complex clinical manifestations of FECD due to incomplete penetrance [1], a deeper understanding of these mechanisms is warranted.

**Figure 1:**
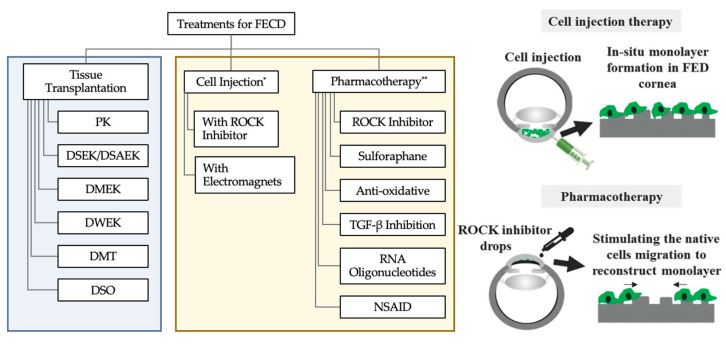
Overview of treatments for FECD, Blue box: Conventional surgical approach, refer to Figure 2. Yellow box: alternative treatment strategies [12,14,18,19,27,35,37,38,41,42,43,44,45,46,47] PK: Penetrating Keratoplasty, DSEK/DSAEK: Descemet Stripping Endothelial Keratoplasty, DMEK: Descemet Membrane Endothelial Keratoplasty, DWEK: Descemetorhexis Without Endothelial Keratoplasty, DMT: Descemet membrane transplantation, DSO: Descemet Stripping Only, ROCK: Rho-associated coiled-coil kinase, NSAID: nonsteroidal anti-inflammatory drugs. The schematic to the right of the yellow box, denoted by * and **, exemplifies cell injection therapy and pharmacotherapy, respectively [48].

Currently, the only option to reverse corneal blindness caused by FECD is surgical intervention, which involves replacing the diseased corneal endothelium with healthy cadaveric tissue [27]. However, there is a severe shortage of suitable donor graft material, and while over 185,000 corneal transplants are performed yearly, an estimated 12.7 million individuals globally are waiting for corneal transplantation [28]. Therefore, the development of alternative treatment modalities is imperative [10,11,28,29,30,31,32]. Indeed, a multitude of drug-based treatments [4,12,18,19,20,21,22,23,24,25,26] have exhibited great potential in mitigating the effects or onset of FECD [4,10,11,12,13,14,15,16,17,18,19,20,21,22,23,24,25,26,27,28,29,30,31,32,33,34,35,36,37,38,39,40,41] (Figure 1). On the surgical front, research has demonstrated the feasibility of reversing corneal blindness through cellular therapeutics using expanded corneal endothelial cells in the form of corneal endothelial cell injection therapy [12,13,14,15,16,17,18].

To develop novel or alternative treatment strategies for FECD, a deeper understanding of its pathology is essential. This requires the establishment of validated FECD disease models that can recapitulate its pathology, progression of pathogenesis, and diseased microenvironment.

The main objective of this review is to summarize the advancements in the development of FECD disease models for preclinical studies. This review is expected to provide further insight into potential therapies for FECD.

## 2. Cornea Anatomy and Functions

**Figure 2:**
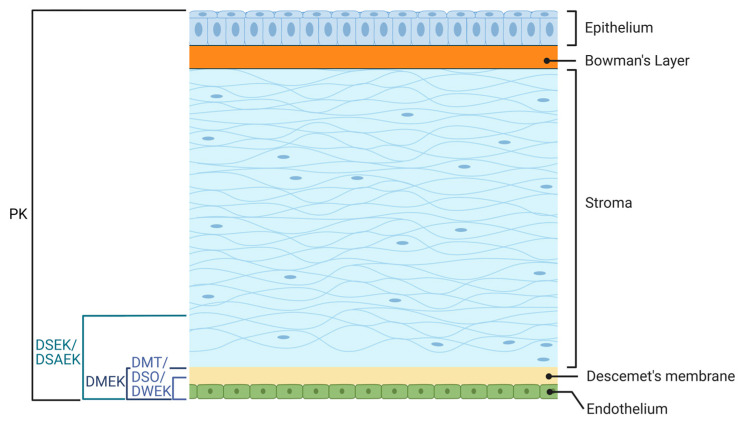
Cornea structure and the layer(s) of tissue removed for each respective surgical procedure (Created in BioRender (www.biorender.com) based on information from [49]).

The cornea is a transparent structure at the front of the eye, comprising five layers: the corneal epithelium, Bowman’s layer, corneal stroma, Descemet’s membrane, and corneal endothelium (Figure 2). The characteristics of these five layers are summarized in Table 1. The main objective of constructing a disease model of FECD is to better understand the normal and aberrant physiologies of the posterior layers of the cornea, particularly the stroma, Descemet’s membrane, and corneal endothelium.

### 2.1. Stroma

The corneal stroma is posterior to Bowman’s layer and constitutes 80–85% of the entire corneal thickness. It comprises highly organized collagen fibers, proteoglycans, and the ECM. Stromal collagen is a heterodimeric complex consisting of type I and V collagens. These collagen fibers are densely packed, forming fibrils that are arranged into highly ordered layers or lamellae with adjacent, parallel fibrils. The human corneal stroma contains between 200 and 250 lamellae that are arranged perpendicularly. This strict organizational pattern allows the cornea to effectively minimize forward light scattering and maintain its mechanical strength [50].

### 2.2. Descemet’s Membrane

The Descemet’s membrane (DM) is an acellular basement membrane of the corneal endothelium, with two distinct structural sections: the anterior banded layer (ABL) and the posterior non-banded layer (PNBL). The anterior layer is formed before birth, while the posterior layer is secreted by endothelial cells (EC) [50].

### 2.3. Endothelial Layer

The innermost corneal layer is the corneal endothelium, a monolayer of hexagonal cells that spans the DM and adheres through numerous hemidesmosomes between the two layers. Corneal endothelial cells are derivatives of the embryonic neural crest and, once established, remain quiescent in vivo [51]. The density of endothelial cells decreases throughout the human lifespan, from approximately 5000 cells/mm^2^ at birth to approximately 2000 cells/mm^2^, gradually over time [52]. This monolayer also flattens from 10 µm at birth to 4 µm in thickness later in adulthood.

The CE plays a crucial role in maintaining corneal clarity by removing fluids from the other layers, thereby maintaining the deturgescence of the cornea. Abundant Na^+^/K^+^-ATPase pumps actively exchange Na^+^ and K^+^ ions across the basolateral membrane, establishing high intracellular K^+^ and extracellular Na^+^ concentrations. The Na^+^ ions pass through the endothelium layer into the aqueous humor, and in the process, draw fluids away from the stroma through passive osmosis. The endothelial layer is unique in that it is permeable to a degree of ion flux sufficient to establish an ionic gradient [50].

**Table 1:** Characterization of the five layers of the cornea.

Structural Name	Prime Characteristic	Cellular/Acellular	Main Function	Developmental Origin	Ref.
**Epithelium**	Formed in three distinct cell layers: superficial cells, wing cells, and basal cells	cellular	Protect against pathogens and toxins, and frontal barrier	Derived from surface ectoderm at 5–6 weeks during development	[50]
**Bowman’s layer**	An acellular layer made up of collagen fibrils	acellular	Provides structural support to the corneal epithelium	Becomes noticeable at 13 and 19 weeks of embryo development	[50,53]
**Stroma**	The bulk of the cornea structure, highly organized	cellular	Provides corneal transparency and mechanical support	During the second wave of neural crest migration (at seven weeks), after primitive endothelium has formed	[50]
**Descemet’s membrane (DM)**	Structurally formed by the anterior banded layer (ABL) and the posterior non-banded layer (PNBL)	acellular	Supports the corneal endothelium structure	The anterior layer was formed before birth at the 8-week stage. Endothelial cells continuously secrete Descemet’s membrane before and after birth	[50]
**Endothelium (CE)**	A monolayer of cells hexagonal in shape that span along the DM	cellular	Keep the clarity of the entire cornea by maintaining the corneal hydration dynamically via a “pump-leak” process	Derived from Neural crest monolayer of cells	[50]

### 2.4. Abnormal Physiology—FECD

In patients with FECD, significant changes occur in the DM and CE layers. Abnormal secretion by FECD CEnCs leads to the formation of characteristic drop-like excrescences known as guttata over an extended period of time [54,55]. The accumulation of these extracellular deposits is associated with the severity of the disease [2,54,56]. Guttata primarily occurs in the center of the DM [53], and as the central corneal endothelium undergoes accelerated cell loss, migration of the peripheral CEnCs towards the central region occurs, leading to polymorphism and polymegathism of the corneal endothelium [57], which also affects cell migration. However, it remains unclear whether the presence of guttata interferes with the re-establishment of functional integrity.

The reduction in CEnCs leads to an overall decrease in the number of available ion pump sites. As the disease progresses to the extent that the loss of CEnCs falls below a critical threshold, the dynamic functional capacity of the corneal endothelium is overcome, affecting the rate of fluid removal from the stroma [1]. Consequently, a cascade of physiological events occurs, including reduced visual acuity and stromal edema [51,58]. If left untreated, this will ultimately result in corneal blindness [2].

## 3. FECD Disease Pathology

**Figure 3:**
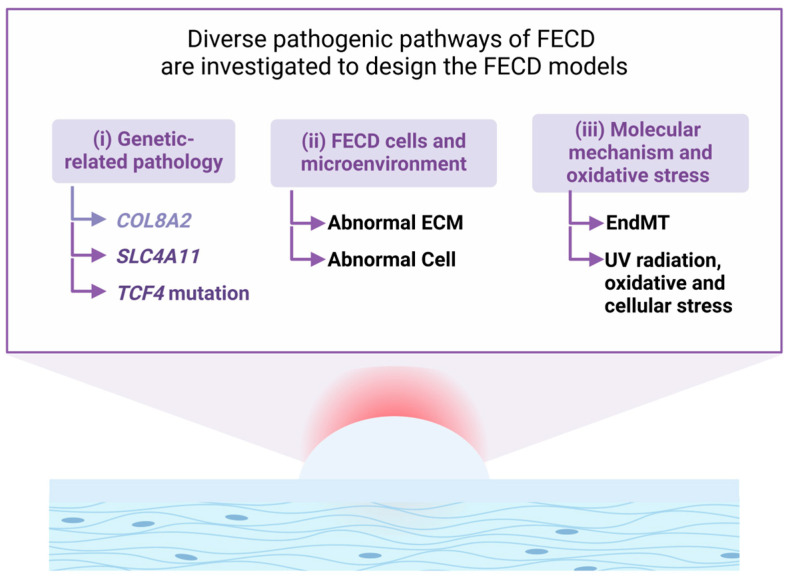
An overview of FECD pathology. Pathology can be further elucidated as (i) genetic-related pathology linked to early onset (*COL8A2*, light purple text), and late-onset (*SLC4A11* and *TCF4* mutations, dark purple text), (ii) FECD cells and the microenvironment, and (iii) molecular mechanisms and oxidative stress. Created in BioRender (www.biorender.com).

Designing an FECD model requires a coherent understanding of both FECD pathogenesis and corneal endothelium physiology (Figure 3). Despite extensive research on the diverse pathogenic pathways of FECD, existing theories have not yet managed to encompass all cases of the disease [1]. Mutations in genes encoding *TCF4*, *COL8A2*, and Solute carrier family 4-member 11 bicarbonate transport protein (*SLC4A11*) genes have been identified. Molecular mechanisms, such as endothelial-mesenchymal transition (EndMT) [59] and guttata cytotoxicity [48,60], have been described, and the currently proposed mechanisms lead to guttata formation and eventually increased endothelial cell apoptosis. A recent study applied gene bioinformatics analysis from the Gene Expression Omnibus database and identified differentially expressed genes, including extracellular matrix related genes, with the top three enriched pathways for cytokine-cytokine receptor interaction, transforming growth factor beta (TGF-β) signaling, and RIG-I-like receptor signaling [61].

### 3.1. Genetic-Related FECD Pathology

Approximately 37–39 percent of patients with FECD exhibit hereditary autosomal dominant mutations [1]. It is believed that various genetic mutations can trigger the development of FECD. For example, *COL8A2* is closely linked to the early-onset form of FECD [62]. Other genes, such as *TCF4* mutation, *SLC4A11*, Zinc finger E-box-binding homeobox 1 (*ZEB1*), lipoxygenase homology domains 1 (*LOXHD1*), and ATP/GTP binding protein-like 1 (*AGBL1*), are more closely associated with the late-onset form of FECD [63,64], with a trinucleotide repeat expansion in *TCF4* being the most prevalent mutation [65]. Interestingly, these genes are involved in a wide array of cellular processes, including ECM deposition, apoptosis, ion transportation, intercellular contact, and cell proliferation. Notably, the clinical manifestations of FECD are genetically heterogeneous, with the expression levels varying among cases, and the penetrance being incomplete among some patient populations [2]. In the subsequent section, the three most common FECD-associated gene mutations are summarized.

#### 3.1.1. COL8A2

The *COL8A2* gene encodes collagen VIII α2-subunit short-chained collagen, which is secreted by CEnCs and deposited on the DM [66]. Mutations in this gene have been linked to early-onset FECD, rather than the late-onset form [63,66]. A specific mutation at L450W of *COL8A2* has been found to increase the deposition of both collagen VIII α1 and α2 in the anterior banded layer (ABL) of the DM, along with deposition of collagen IV, fibronectin, and laminin, tripling the average thickness of the normal DM [67]. Another mutation, specifically Q455K, showed early-onset FECD traits in mouse models without displaying a severe FECD phenotype [11,68]. Jun et al. proposed that this mutation might be related to endoplasmic reticulum (ER) stress and activation of the unfolded protein response (UPR). Another study suggested that the DM was of lower stiffness compared to wild-type controls, potentially hindering CEnC maintenance and function [68]. Although there are currently several working theories, the precise mechanism underlying the association between *COL8A2* mutations and FECD remains elusive.

#### 3.1.2. SLC4A11

SLC4A11 is a member of the SLC4 bicarbonate transporter family of proteins [29] and has been associated with both dominant late-onset FECD and recessive congenital hereditary endothelial corneal dystrophy type 2 (CHED2) [69]. Positioned in the basolateral membrane of CEnCs, it facilitates the transport of ions and water flux across the membrane, including NH_3_, OH^−^ and H^+^ ions. It has also been suggested that SLC4A11 acts as a cell adhesion molecule in DM [70]. The FECD missense mutation does not affect intracellular trafficking of the protein, implying that the mutant phenotype stems from altered channel functionality [29]. Other studies have also implied that mutations in the *SLC4A11* gene are associated with the misfolding of proteins, resulting in the generation and accumulation of reactive oxygen species (ROS), leading to mitochondrial dysfunction due to oxidative stress [71]. In addition, SLC4A11 has been suggested to be involved in the adhesion of CEnCs [70]. However, the key contributors of missense *SLC4A11* mutations that result in the different variants of corneal dystrophy described remain unclear.

#### 3.1.3. TCF4 Mutation

The *TCF4* gene is located on chromosome 18 and encodes transcription factor 4 protein. It has been reported that an intronic non-coding cytosine–thymine–guanine (CTG) trinucleotide repeat (TNR) expansion within this *TCF4* gene, termed CTG18.1, is associated with FECD [31]. The primary proposed pathogenic mechanism involves CTG repeats forming transcript aggregates in the nucleus, sequestering muscle-blind-like 1 protein (MBNL1), an essential protein in messenger RNA (mRNA) splicing, leading to downstream mis-splicing events in other important mRNAs [72,73]. The differentially spliced genes that result from this process were found to be enriched for products that localize to the cell cortex and bind to cytoskeletal and cell adhesion proteins [74]. Notably, 79% of patients with FECD phenotype in a US-Caucasian cohort exhibited CTG repeat expansion [65], with similar findings reported in cohorts with Northern European ancestry [75]. However, CTG TNR-associated FECD in Asian ethnicities showed a relatively lower prevalence, with 26% in the Japanese cohort [76] and 44% in the Chinese population in Singapore [77]. It should be noted that such variations in cohorts add a layer of complexity to the relationship between FECD and *TCF4* mutation. Furthermore, it has been reported that the corneal endothelial cells in FECD were reported to become mesenchymal-like cells [78]. The observed endothelial to EndMT may be associated with *TCF4* as it encodes the E-protein family (E2-2), which is closely associated with EndMT (see Section “Endothelial-to-Mesenchymal Transition (EndMT)”).

Interestingly, CTG expansion is more highly expressed in corneal endothelial cells than in cells from other corneal tissues. Examining the CUG repeat RNA foci in different parts of the anterior cornea, the CUG repeat is less detectable in trabecular meshwork cells, keratocytes, or corneal epithelium and absent in lens epithelium, but significantly higher in endothelial cells [79].

A recent study employing transcriptomic techniques in 31 patients with FECD found two distinct pathogenic mechanisms: RNA toxicity and TCF4 isoform-specific dysregulation [80]. Other researchers have attempted to use immortalized FECD human CEnCs (HCEnCs) with TCF4 CTG repeats to identify potential treatments for FECD, such as CRISPR-dead Cas9 [81] and antisense oligonucleotides [82,83]. Further understanding of this will enable the development of new therapies.

### 3.2. FECD Cells and Microenvironment

While 37–39 percent of FECD patients exhibit familial genetic linkage (genes closely located on the same chromosome and inherited across generations), the majority of cases are sporadic [1]. However, the general clinical manifestations of most forms of FECD include abnormal cell behavior, impaired CE function, and apoptosis. The central underlying question revolves around whether cell death and loss of function arise from cell dysfunction, loss of pump function, or the formation of an abnormal microenvironment. Consequently, these pathological events have led to the exploration of mechanisms such as EndMT, cell senescence, cell apoptosis, and the ECM guttata microenvironment as potential mechanisms in FECD pathogenesis.

#### 3.2.1. Abnormal ECM (Guttata)

Aberrant secretion of ECM leads to guttata formation, a crucial characteristic of vision-impairing FECD. The impact on cell function is contingent upon the presence of guttata, which is based on their size and density. In 2016, Rizwan et al. found that synthetic guttata pillars with 20 μm diameter, spacing, and height, as well as 40 μm diameter, could disrupt primary CEnC monolayer formation [48]. However, guttata of smaller heights and lower density did not impede monolayer formation. Similarly, Kocaba et al. observed a comparable outcome in CEnC response to guttata size variations within ex vivo tissue samples [60]. In addition, they found that guttata did not trigger intercellular pathologies, such as DNA damage, defective ROS response, mitochondrial dysfunction, or UPR. Thus, the presence of guttata alone may not fully represent the characteristics of FECD pathogenesis.

#### 3.2.2. Abnormal Cell

##### Endothelial-to-Mesenchymal Transition (EndMT)

The main characteristics of FECD include gradual thickening of the DM due to secretion from diseased cells and the formation of guttata. The pathological mechanism underlying these features remains poorly understood; however, it has been associated with EndMT, a variant of epithelial-to-mesenchymal transition (EMT) [59], potentially involving TCF4 TNR and E2-2 [78]. EndMT involves the transition of endothelial cells from an endothelial to a fibroblastic phenotype, resulting in the destabilization of cell junctions and loss of apical-basal polarity, leading to CEnC motility changes [45,71]. EndMT also leads to the disposition of excessive ECMs, such as collagen I and III, fibronectin, and TGF-β [45]. It has been suggested that activation of TGF-β signaling drives the accumulation of ECM components through activation of EndMT inducer genes such as ZEB1 and Snail1. When found in high expression levels in FECD CEnCs, it leads to the overproduction of ECM proteins like Collagen type I and fibronectin [59]. It has also been proposed that alternative pathways (i.e., FGF-2) could regulate the expression of ZEB1 and Snail1 [84].

##### Cell Senescence

An indication of cell senescence is the loss of the proliferative capacity. While senescence is a regular programmed process in healthy cells during physiological development, it has been implicated in chronic diseases such as FECD. It has been suggested that the interaction between FECD CEnCs and abnormal ECM is associated with premature cellular senescence and cell death through cellular aging [2].

Researchers have speculated that premature cell senescence may be related to the pathogenesis of FECD. Studies by Matthaei et al. [85] and White et al. [86] revealed that *COL8A2* knock-in mouse models and oxidative stress induced by exposure to UVA radiation led to senescence of CEnCs. This was evidenced by the significant upregulation of the gene encoding cyclin-dependent kinase inhibitor 1A protein (CDKN1a, also known as p21, related to cell cycle arrest) and elevated p53 levels. This led to a series of proposed mechanisms associated with senescence: persistent DNA damage could lead to CEnCs arrest in the G2/M stage, causing premature cell senescence. This, in turn, could induce a profibrotic phenotype involving abnormal ECM secretion and EndMT in CEnCs, as observed in FECD [86].

#### 3.2.3. Oxidative Stress and EndMT

Oxidative stress is believed to play a crucial role in the pathogenesis of FECD [2], as the cornea is constantly exposed to UV light and high oxygen level metabolic activity throughout life [2,87]. In normal CEnCs, when an excessive amount of ROS is present, oxidative stress activates a series of cellular defense mechanisms, such as initiating the production of antioxidants and apoptosis pathways [2]. However, in FECD cells, these pathways may be diminished due to the abnormal aging process [88], as evident from the downregulation of antioxidant-associated transcripts (peroxiredoxin 2 (PRDX2), PRDX5, and superoxide dismutase 2 (SOD2)), the upregulation of an oxidative DNA damage marker, 8-hydroxy-2′-deoxyguanosine, within the FECD endothelium [88,89], and ferroptosis [90,91,92].

Katikireddy et al. employed a combination of TGF-β and menadione (MN) on immortalized HCEnCs to investigate oxidative stress’s role in FECD pathogenesis. Their study showed that MN could induce EndMT and that the impact of ROS levels might be further amplified by the absence of NQO1, which is responsible for shielding against oxidative stress [44]. Azizi et al., through experiments with FECD and healthy human corneal endothelial cell lines exposed to tert-Butyl hydroperoxide (tBHP), demonstrated that the FECD cell line displayed heightened susceptibility to oxidative stress-induced apoptosis and DNA damage, coupled with heightened p53 protein activation [93].

Ferroptosis is the predominant mechanism in FECD pathogenesis. Although researchers have focused more on apoptosis, ferroptosis may interconvert or work with apoptosis to cause cell death [92]. Ferroptosis is regulated by several metabolic pathways, including fatty acid metabolism, iron handling, and mitochondrial function [92]. Lovatt et al. found that the loss of PRDX1 protein expression in CEnCs due to increased oxidative stress causes lipid peroxidation and leads to ferroptosis [94]. Their study also provided evidence that the loss of the transcription factor nuclear factor erythroid 2-related factor 2 (Nrf2), a vital transcription factor for antioxidant defense, can increase lipid peroxidation in CEnCs [94].

## 4. Models Used to Study FECD

**Figure 4:**
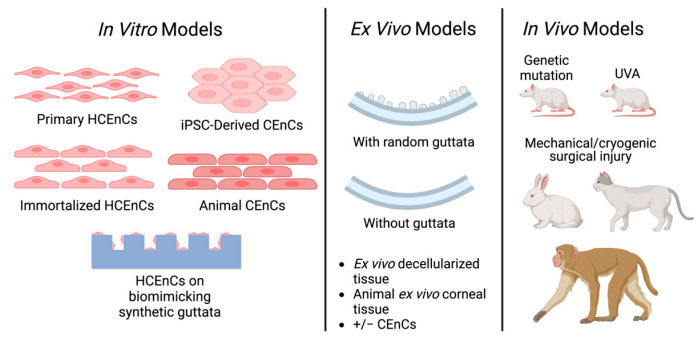
Various models have been used to study FECD. For in vitro models, primary HCEnCs, immortalized cell lines, animal CEnCs, and HCEnCs on biomimicking synthetic guttata have been established. For ex vivo models, human ex vivo decellularized tissues and animal ex vivo corneal tissues are used. For in vivo models, genetic mutation and UVA-induced murine models, as well as rabbit, feline, and cynomolgus monkey models created by mechanical or cryogenic surgical injury, are commonly used. Created in BioRender (www.biorender.com).

The development of a disease model must encompass a thorough process of exploration based on the understanding of the disease’s molecular mechanisms and microenvironment to reflect the intricacies of its etiology. This section will delineate both the strengths and limitations of some of the current FECD models (Figure 4), including in vitro models with primary and immortalized cell lines, ex vivo models, and in vivo models, offering insights into potential methodologies for improvement.

### 4.1. In Vitro Models

In the wake of major advancements in tissue engineering [95], the conceptual goal of building a functional full-thickness cornea has gained traction since the late 20th century, aiming to revolutionize ophthalmic drug screening, minimize reliance on animal models, and offer the enticing prospect of alternative solutions for corneal transplantation [96]. Alongside this ambitious pursuit, various simple ECM-supported two-dimensional cell culture models with primary or immortalized healthy or FECD CEnCs have been developed to study FECD (Table 2).

#### 4.1.1. Primary Human CEnC Models

##### Primary Human FECD CEnCs

Studies on early embryogenesis have shown that the human corneal endothelium originates in the neural crest [118]. However, the regenerative capacity of the human corneal endothelium in vivo is extremely limited. Additionally, the propagation of CEnCs in vitro has historically been challenging, particularly in maintaining their characteristic polygonal morphology and preventing EndMT [119,120]. While not entirely resolved, specific culture techniques, such as the dual media culture system [100] and the utilization of substrate support [101,102], have shown promise in addressing these obstacles. Despite limitations in their propagation profiles, the characteristic phenotype of the propagated primary CEnCs can be maintained [97], making them the closest model to their in vivo counterparts [103].

The use of CEnCs isolated from FECD patients has primarily been utilized to study cell propagation and behavior [16,98,99]. Zaniolo et al. harvested central FECD cells, mostly at advanced stages, from 29 patients. While cell cultures were successfully established in 18 samples, cell morphology varied greatly among donors, with six samples showing fibroblastic-like elongated cell morphology, atypical of CEnCs. FECD CEnCs from patients without a Descemet’s membrane fibrillar layer and younger patients (<30 years) showed higher success rates for both culture establishment and maintenance of endothelial morphology [98]. It is worth noting that cells from younger patients have a better proliferative capacity compared to those from older donors. These findings suggest that age and the presence of the fibrillar layer could be valuable predictors for a successful culture, and in a conducive environment, CEnCs isolated from FECD DM still possess the proliferative ability for in vitro culture [98].

It should be noted that primary FECD cells in vitro have been observed to undergo a phenomenon of “phenotypical switching”, where they seem to regain the typical healthy features of CEnCs, such as restored telomere length, mitochondrial DNA (mtDNA) level, and balanced antioxidant levels [98,99].

However, primary FECD cells, when cultured in vitro, have been observed to restore a normal phenotype with similar mtDNA levels, telomere length, oxidant-antioxidant gene expression balance, and sensitivity to oxidative stress-induced cell death, compared to healthy cells [99]. While such reports complicate the understanding of the disease mechanisms of FECD, a likely explanation for the phenotypical switch may be the selection pressure of in vitro culture. Such culture environments are generally nutrient-rich and formulated to provide conducive conditions for cellular proliferation, which favors cells with healthy characteristics, and in turn, enables the healthier cells to thrive and “out-grow” the disease FECD cells, hence “restoring” the normal phenotype following propagation [98,99].

In addition to developing in vitro FECD CEnCs cell culture methods and the characterization of primary FECD cells, studies have also focused on the development of novel treatment strategies for FECD through the use of regenerative cell therapy, and gene therapy targeting FECD genetic mutations. For instance, based on the earlier work of McGowan et al. [121], neural crest-derived progenitor (NCDP) cells have been identified within both healthy and FECD primary CEnCs, with vast propagation and differentiation capacities into neural crest derivatives [97]. The identification of these NCDP cells undoubtedly opened a possible opportunity for autologous cell therapy for FECD, but more studies need to be conducted as the directed differentiation of these NCDP back into functional CEnCs remains elusive. Conversely, as the occurrence of FECD is largely associated with an intronic CTG repeat expansion in TCF4, the utilization of gene-silencing technology, such as antisense oligonucleotides, to target the TCF4 TNR expansions within FECD primary CEnCs to suppress the expression of these CTG repeats to mitigate RNA toxicity has been described [41], thereby opening a plausible gene-based strategy for FECD treatment.

Consolidation of the current understanding revealed that normal primary CEnCs can be isolated and propagated from cadaveric donor corneas. With the isolation of primary CEnCs from FECD donors, it has been shown that isolated FECD cells tend to form both endothelial and fibroblastic morphologies in culture [98], and novel autologous cell therapies have also been developed and analyzed [41,121]. However, considerations for the propagation of primary FECD cells must be addressed as additional challenges when compared to the culturing of non-FECD primary CEnCs. Specifically, FECD patient samples are generally obtained from older patients with corneas with low corneal endothelial cell counts who are undergoing corneal transplantation. The extraction of these FECD samples during surgery may further decrease the overall cellular count and viability of the FECD sample. Taken together, the subsequent isolation of viable FECD cells may hence be of poorer overall cell yield, resulting in a non-optimal culture condition where the initial seeding density may affect the proliferative propensity of these cells [122].

#### 4.1.2. Immortalized Human CEnCs

While the propagation of primary CEnCs has been well described [123], the isolation of primary CEnCs from patients with FECD remains a challenge [124]. While the utilization of FECD-derived immortalized CEnCs offers a source of cellular material for preliminary FECD research, bypassing the challenges of primary FECD cell culture, it should be noted that their altered behavior and responses raise concerns [104,105,106,107], particularly in studies involving cell proliferation and drug effects [48]. These immortalized lines may exhibit phenotypic changes [104,105], with partially impaired pump functions [106,107], and/or contain varying gene and protein expression profiles compared to each other and to primary CEnCs [103]. Despite these limitations, immortalized CEnCs remain widely used for in vitro FECD modelling, particularly as a pre-screening tool before committing to the use of primary cells [48]. The subsequent sections summarize the derivation and use of immortalized primary CEnCs and primary FECD cells.

##### Immortalized CEnC Lines from Primary HCEnCs

Immortalized CEnCs, such as SV40 (simian virus) large T-antigen-induced cell lines, were the first viral oncogenes used to immortalize human CEnCs [104]. The survivability of CEnCs from both young and old donors improved after transfection. However, SV40 cell lines have distinctive characteristics that are not observed in normal HCEnCs. For instance, cells may become stratified due to overconfluence [104]. The HCEnC-12 cell line also shows differences in gene expression when compared to primary CEnCs [103].

In addition to the large T-antigen, the SV40 small T-antigen offers an alternative approach to immortalization. Valtink et al. described the resultant HCEnC-12 cell line as having a preserved hexagonal morphology similar to that of primary CEnCs. Notably, further subcloning led to two distinct lines: HCEnC-B4G12, closely recapitulating primary CEnCs in vivo with their monolayered polygonal morphology, and HCEnC-H9C1 possessing characteristics of CEnC-like cells in an earlier developmental or transitional stage [104,105]. Using the HCEnC-12 cell line, Jurkunas et al. demonstrated an oxidative imbalance, as well as increased oxidative DNA damage in FECD CE compared to normal CE, a phenomenon observed in both donor tissues, as well as in SV40-transfected CEnCs under oxidative stressed conditions in sub-confluent cultures [88]. Consistent with their observations in CE, immortalized CEnCs showed decreased cell viability and downregulation of Nrf2, along with increased levels of DNA oxidation upon treatment with hydrogen peroxide (H_2_O_2_). This instance highlights the use of SV40-immortalized CEnCs to provide valuable molecular insights that complement the analyses of human CE samples [88].

Human papillomavirus (HPV) type 16 transforming oncoproteins E6 and E7 and Cdk4R24D/CyclinD1 were used to create two transduced CEnC cell lines with longer lifespans [107]. The Cyclin-transduced CEnCs showed hexagonal morphology, whereas the E6/E7-transduced HCEnCs could not maintain their hexagonal shape. The mRNA expression of ZO-1 and N-cadherin proteins was detected in both Cyclin-transduced and E6/E7-transduced cell models; however, ZO-1 and N-cadherin proteins could only be immunostained at the intercellular junction in Cyclin-transduced HCEnCs. Subsequent tests also showed reduced pump function in Cyclin-transduced HCEnCs. The difference was speculated to be caused by the post-translational import of the ZO-1 protein. Despite its limitations, E6/E7-transduced HCEnCs are valuable for studying the impact of environmental UVA radiation on Nrf2-regulated antioxidant levels [108]. However, a potential but crucial drawback lies in the oncogenes’ ability to inhibit tumor suppressor genes like p53, and this will interfere with any FECD studies on p53-mediated apoptosis [93].

Unlike the HPV E6/E7 oncogene immortalization approach, the use of human Telomerase Reverse Transcriptase (hTERT) immortalization introduces the hTERT gene into CEnCs to telomerase activity [110]. By selecting a unique subpopulation of proliferative primary CEnCs and genetically introducing hTERT, the HCEnC-21T cell line showed high proliferation with minimal senescence and no signs of an oncogenic phenotype, while maintaining many of the key characteristics of primary CEnCs, such as the levels of ion transporters and barrier integrity [110].

The established HCEnC-21T cell line provides an avenue for better understanding the role of oxidative stress in EndMT and cellular remodeling in patients with FECD. Katikireddy et al. investigated whether MN, a molecule known to induce oxidative damage, could trigger EndMT in FECD [44]. By generating NAD(P)H quinone oxidoreductase 1 (NQO1) knockout (NQO1^−/−^) cells from the HCEnC-21T line, effectively removing the enzyme that breaks down MN, and comparing the effects of MN on both regular and NQO1-deficient HEnC-21T, it was found that the absence of NQO1 amplified the severity of MN-induced EndMT, suggesting a protective role against oxidative stress and potentially driving FECD progression [44]. Qureshi et al. also used the HCEnC-21T cell line to study endoplasmic reticulum (ER) stress on immortalized HCEnC-21T cells by ER stressor tunicamycin [125]. Tunicamycin-induced ER stress disrupts mitochondrial function, resulting in the loss of CEnC viability and downregulation of the anti-apoptotic Bcl2 in HCEC-21T cells and in ex vivo corneal tissue.

Immortalized corneal cells have become a useful model for testing oxidative stress and potential antioxidants in FECD. Ceravolo et al. applied 200 μM H_2_O_2_ to induce FECD-mimicking oxidative stress in immortalized human corneal epithelial cells (HCEpCs, Epithelial Adenovirus 12-SV40 hybrid transformed HCE cells) [126]. They showed that the presence of Aloe vera extract (100 μg/mL) reduced oxidative stress-induced inflammatory responses and apoptosis. The same group of researchers also applied H_2_O_2_ treatment to immortalized HCEnCs as an FECD model to study the anti-inflammatory effect of polydeoxyribonucleotide (PDRN) [127]. They found that PDRN helped protect HCEnCs from H_2_O_2_-induced damage and that adenosine was involved in PDRN actions.

##### Immortalized FECD Cell Line

Immortalized FECD cell lines (iFECDCs), despite being valuable tools for studying FECD, have limitations. While in vitro expanded iFECDCs exhibit the formation of contact-inhibited polygonal monolayers, similar to immortalized HCEnCs, FECD cell lines often exhibit subtle differences from their primary-cell counterparts [59,113].

One of the earliest studies describing the immortalization of FECD CEnCs was reported in 1997, where He et al. transduced FECD patient CE using a disabled HPV E6/E7-carrying retrovirus. The resultant transduced FECD CEnCs were similar to those of the transduced normal CEnCs. Surprisingly, the immortalized FECD cells displayed comparable morphology, protein patterns, and proliferation rates to normal E6/E7-transduced cells, suggesting a remarkable resemblance despite the underlying quantitative protein differences [106].

Using SV40 large T-antigen and hTERT, Okumura et al. described the immortalization of CEnCs from healthy donors (iHCEnCs) and FECD patients (iFECDCs) [59]. Notably, iFECDCs exhibited distinct features compared to iHCEnCs, with significantly upregulated EndMT-related genes and excessive production of fibrillar ECM, both between and within cells, suggesting aberrant cellular remodelling in FECD [59]. In a subsequent study, Okumura used both iHCEnCs and iFECDCs cell models to support their observation that the expression levels of TGF-β isoforms and their receptors were higher in the CE of FECD patients. In iFECDCs, upregulated UPR and apoptotic signals upon TFG-β treatment, suggest a link between the TGF-β pathway and FECD development [45].

Jurkunas et al. (2010) took the approach of generating immortalized iFECDCs, as well as HCEnC-21T from healthy donors using SV40 T antigen (as mentioned in Section “Immortalized CEnC Lines from Primary HCEnCs”), to elucidate the mitophagy pathways, a cellular process involved in mitochondrial recycling [88]. Interestingly, iFECDCs exhibited a significant decrease in mitochondrial mass due to autophagy, suggesting a process that is dysregulated in FECD. They further discovered the downregulation of mitofusin 2 (Mfn2), a protein important for mitochondrial function and antioxidant balance, potentially contributing to FECD pathogenesis [114]. A subsequent study by Ong Tone et al. (2021) revealed that iFECDCs displayed protein expression and morphological differences compared to iHCEnCs. Specifically, iFECDCs were able to retain the TCF4 CTG mutations of the patient’s cells after immortalization [113], and these iFECDCs were found to be migratory and fibroblastic in morphology [113]. Using one of the cell lines established by Ong Tone et al., Yan et al. observed iFECDC migration in an EMT-independent manner, with altered microtubule stability which promotes cell migration in iFECDCs [128]. The iFECDC cell lines have been used to study the role of Ataxia-Telangiectasia Mutated (ATM), a serine/threonine kinase belonging to the phosphatidyl inositol 3-kinase (PI3K) family, in oxidative stress-induced DNA damage and senescence [129]. They applied UVA as a physiological stressor, MN as a chemical stressor, and catechol estrogen to iHCEnCs and iFECDCs. While acute stress increases cell-cycle arrest and DNA repair in the G2/M phase, chronic stress with UVA and catechol estrogen induces ATM-driven cell-cycle arrest in the G0/G1 phase, reduces DNA repair, and induces cytotoxic senescence. These results were also validated in an ATM-knockdown mouse model, providing some mechanistic understanding of the role of ATM in oxidative stress disorders like FECD.

Another immortalized FECD cell line, F35T, derived from a 62-year-old female FECD patient expressing *TCF4* transcript with approximately 4500 CTG repeats in the intron region [91,130], has been used in multiple studies, such as trinucleotide repeat-targeting CRISPR-dead Cas9 (dCas9) as a potential treatment for FECD [81], antisense oligonucleotides to target *TCF4* expansion by targeting mutant-repetitive RNA [82], and tissue-specific *TCF4* triplet repeat instability using optical genome mapping [130]. Recently, Saha et al. used the F35T *TCF4* trinucleotide repeat expansion cell line with UV irradiation to study ferroptosis [91]. They found that UVA and RSL3, an inhibitor of GPX4 peroxidase activity and inducer of ferroptosis, could induce ferroptosis more significantly in F35T cells than in control B4G12 cells. The results were also validated using ex vivo FECD and healthy corneal tissues.

#### 4.1.3. Induced Pluripotent Stem Cells—Derived CEnCs

Induced pluripotent stem cells (iPSCs)-derived cells have been used for disease modeling, drug development, and cell therapy [131]. In order to overcome the disadvantage of using immortalized cell lines, which cannot evaluate the precise mechanisms of cell death, and the challenges of isolating and culturing primary FECD CEnCs, Sakakura et al. developed an in vitro FECD model by inducing CECs from disease-specific iPSCs of FECD patients [115]. They found that compared to immortalized HCEnCs, iPSC-derived HCEnCs were more susceptible to cell death and may more accurately reflect pathophysiology. In another study, Brejchova et al. used iPSC-derived CE-like cells to study whether disease-associated variants induce aberrant SLC4A11 pre-mRNA splicing, which is a dominant genetic-related pathology of FECD [132]. However, challenges remain, including high generation and maintenance costs, the presence of residual undifferentiated iPSCs, and limited reproducibility, which need to be further explored [116].

#### 4.1.4. Primary HCEnCs on Biomimicking Synthetic Guttata

The biomimicking synthetic guttata model is an in vitro model constructed based on clinical observations of the guttata microenvironment. By biomimicking the disease microenvironment, monolayer formation, cellular migration, and cell expansion can be better understood.

Rizwan and colleagues used polystyrene to synthesize an array of microstructures mimicking the guttata in the FECD microenvironment. They created a guttata model with various dimensions and structures resembling guttata at different stages of FECD [48]. These ranged from 10 to 80 µm in guttata diameter, spacing between nodules, and height, with shapes of either pillars or domes. This study provides insights into the engineering of in vitro FECD models with controlled dimensions and shapes.

To validate the CEnC monolayer reformation ability of the FECD DM after pharmacotherapy using the ROCK inhibitor with cell injection treatment, they initially used HCEnC-B4G12 cells for a preliminary study. Subsequently, they expanded primary CEnCs on the synthetic guttata model to investigate monolayer formation and found that when the height, diameter, and spacing of the guttata reached 20 μm, the seeded primary CEnCs failed to form a monolayer [48]. Samples with larger diameters (40 μm) also showed similar results. Interestingly, patterns with lower heights showed a higher rate of monolayer recovery; this indicates that the diameter, spacing, and height of the guttata could significantly affect the CEnC monolayer recovery, suggesting that the patient’s recovery after pharmacotherapy and cell injection therapy would be more effective in early stage FECD patients but may not be as successful for late-stage FECD patients with more severe and/or larger guttata. Regarding cell migration, dome structures were shown to improve the migration speed and reduced the directionality of CEnCs compared to pillar structures. [48]. Other synthetic materials, such as gelatin methacrylate (GelMA) and other hydrogels, can also be used to recapitulate the DM microenvironment in vivo with varying stiffnesses [117].

#### 4.1.5. Animal Cell Culture Models

Animal CEn cells are an alternative approach for studying FECD. For example, bovine corneal endothelial (BCE) cells [46] are relatively easy to obtain, but they differ physiologically from human CEnCs. Kim et al. utilized BCE cells for drug screening, specifically to assess UPR and oxidative stress response [40]. To induce UPR and oxidative stress, the extracted BCE cells were treated with thapsigargin or H_2_O_2_. It is known that oxidative stress contributes to EndMT, excessive ECM secretion, and rosette formation in FECD cells [44,46]. However, the authors acknowledged the limitations of using bovine cultures and consequently opted for immortalized human CEnCs to evaluate the survival of HCEnCs after drug treatment. Similarly, Teo et al. conducted preliminary studies on CEnC response to nanotopography using BCE [133]. The research group later validated their studies using immortalized [134] and primary HCEnCs [101]. While the availability of BCE cells would be advantageous for preliminary studies, immortalized human CEnCs are more suitable and consistent representations of healthy human CEnCs [46]. BCE were cultured as spheroids in a three-dimensional environment to study their injectability [135]. Guo et al. found that BCE spheroids had higher stemness potential in vitro and showed better status and greater cell growth adherence when cultured with the ROCK inhibitor Y-27632, compared to BCE cultured in a two-dimensional plate. There are limited studies that use organoids. Anatomically, as CE is a monolayer of CEnCs on the DM, it is sensible that most models, whether in vitro or ex vivo, are developed with a monolayer of CEnCs on two-dimensional culture or three-dimensional guttata structures, instead of employing a multi-cellular spheroid or organoid system. Nonetheless, organoid cultures of ex vivo corneal tissue or DM have been used to study FECD (see Section 4.2).

Another animal CEn cell source for in vitro models is the primary porcine (pig) CEnCs [136,137,138]. For example, porcine CEnCs were used to demonstrate the stimulatory effects of the ROCK inhibitors Y-27632 and H-1152 [138]. The administration of the ROCK inhibitor resulted in the rearrangement of the porcine CEnC cytoskeleton, and enhanced cell migration and proliferation, which is beneficial for wound healing. Moreover, H-1152 demonstrated a notable increase in the migratory and proliferative capabilities of porcine CEnCs in vitro compared to untreated and treated with Y-27632 cells. Applying H-1152 topically also resulted in a substantial reduction in corneal edema and the development of multinucleate CEnCs in vivo, indicating a proliferation process associated with healing. This study demonstrated the potential application of porcine CEnCs for in vitro testing of pharmaceutical compounds. Researchers also used porcine cornea and DM as ex vivo study models (see Section 4.2.2).

### 4.2. Ex Vivo Corneal Tissues

To understand how the microenvironment affects CEnC behavior, ex vivo models have been developed to recapitulate the phenotypic changes and progression of FECD DM and the endothelium. Various ex vivo models have been utilized to study FECD, and in these models, cell migration, cell-cell interaction, cell death, wound healing, and other investigations have been conducted. The current ex vivo models used in FECD research are summarized in Table 3.

#### 4.2.1. Ex Vivo Corneal Tissue

##### Human Ex Vivo Corneal Tissue

Human ex vivo corneal tissues offer the closest representation of corneal endothelial cell-DM interactions. However, their availability is severely limited by the same scarcity issues that affect cadaveric donor corneas. Furthermore, these tissues are restricted to single-use experimental studies, and since high-quality donor corneas are primarily allocated for corneal transplantation, the supply of specimens for ex vivo research is further constrained [2,28]. Despite these limitations, ex vivo corneal tissues have been widely used to study oxidative stress, cell apoptosis, cell migration, and surgical procedures in FECD [37,38,113,139,144] and are often used to validate findings from in vitro cellular studies.

Halilovic et al. used normal and FECD ex vivo corneal tissues to investigate cell morphology and oxidative stress. They observed higher levels of oxidative stress-induced DNA damage in iFECDCs (discussed in Section “Immortalized FECD Cell Line”) and ex vivo FECD samples than in immortalized CEnCs and ex vivo corneal tissue cells. They then compared the effects of MN on normal immortalized CEnCs. When normal CEnCs were exposed to oxidative stress in vitro, they exhibited a rosette-like phenotype similar to that observed in FECD ex vivo specimens. Further analysis revealed that MN-treated immortalized CEnCs also showed oxidative damage to mtDNA and nuclear DNA (nDNA), mitochondrial fragmentation, and cellular apoptosis, consistent with observations made in FECD samples [38].

Cell apoptosis in FECD was studied by Ziaei et al. [37] using ex vivo FECD corneal tissues to study Nrf2 as an antioxidant defense mechanism. tBHP was used to induce oxidative stress in both normal and FECD ex vivo corneas. FECD cells had a deficiency in Nrf2 defense, while sulforaphane (SFN) could enhance the nuclear translocation of Nrf2 and reduce cell apoptosis. Furthermore, the researchers also found that the cell density in ex vivo FECD tissue was limited. Hence, the subsequent study of the SFN mechanism was not performed in tissue samples but in cell lines instead; this implies the possible limitations of the FECD ex vivo model [37]. A recent study also utilized human corneal endothelial explants from patients with late-stage FECD to study the correlation between guttata, mitochondrial markers and oxidative stress levels [145]. A positive correlation was found between the presence of guttata and mitochondrial calcium level and cell apoptosis, while a negative correlation was found between the presence of guttata and mitochondrial mass, membrane potential, and oxidative stress. This study suggests that guttata negatively affect nearby CEnCs by influencing mitochondrial function and inducing apoptosis. Ex vivo tissue can also be a useful tool for validating in vitro studies on cell apoptosis. For example, Qureshi et al. used ex vivo tissue to validate the studies of ER stress on immortalized HCEnC-21T cells using the ER stressor tunicamycin [125]. Their study on ex vivo corneal tissue also showed tunicamycin-induced ER stress affected mitochondrial functions and apoptosis, similar to the observation in immortalized HCEnCs (Section “Immortalized CEnC Lines from Primary HCEnCs”). Saha et al. used healthy and FCED ex vivo samples to validate their in vitro studies with immortalized FECD and normal cells, showing increased level of cytosolic ferrous iron and lipid peroxidation in FECD tissue, compared to healthy controls [91] (Section “Immortalized FECD Cell Line”).

Cell migration is crucial for the FECD disease fate, which can also be studied using ex vivo models. Ong Tone et al. used ex vivo models to study the importance of cell migration in the progression of FECD [113]. They utilized both healthy and FECD (with guttata) human corneal tissue. Firstly, they collected DM with the endothelial layer from donor corneas from FECD and healthy individuals. The specimens were then transfected with green fluorescent protein (GFP) using lipid nanoparticles (LNP), and the mean migration speed of the cells was calculated using an automated live cell tracking system. CEnCs in the FECD sample showed a higher mean migration speed (for low cell density areas) than normal CEnCs [113]. The cells in FECD specimens also migrated in a single-cell manner instead of “monolayer spreading”, which describes the concerted movement of adjacent cells closing the wound [113,144]. Yan et al. used ex vivo tissue to validate the cell migration studies of iFECDCs (Section “Immortalized FECD Cell Line”), showing increased expression of TUBB4A (tubulin beta 4A class IVa), and changes to other cytoskeleton proteins, which affect the iFECD cell migration [128].

Ex vivo models are also useful for surgical method development; for example, they can be used to predict the age of the patient as a factor to decide if a ROCK inhibitor will be needed to promote cell migration after DSO [146], or to evaluate the potential FECD treatment using laser ablation on FECD ex vivo DM [139]. The laser can ablate the diseased tissue guttata on the ex vivo FECD DM. After the surgical procedure, DM and cells were expanded in a medium supplemented with the ROCK inhibitor ripasudil. The resulting cell migration and expansion led to the initiation of wound closure, which was nearly complete after 26 to 38 days. This study, using FECD DM as a model, demonstrated a potential solution for treating abnormal ECM in FECD DM [139]. A recent study also utilized an ex vivo model to study cell migration in response to ripasudil, a ROCK inhibitor, after DSO treatment [43]. The ripasudil-treated immortalized normal, and FECD cells showed significantly enhanced migration. The ex vivo model of normal and FECD DM also showed enhanced cell migration from the DN to the stroma when treated with ripasudil.

##### Ex Vivo Decellularized Tissue

To further study the pathogenesis role of guttata in FECD, researchers used FECD ex vivo tissue without cells. Kocaba et al. developed a combinatorial model by seeding an immortalized cell line HCEnC-21T [60] on decellularized FECD ex vivo tissues. They found that small-diameter (10.5 μm) guttata did not inhibit the proliferation of CEnCs. Whereas medium diameter sized guttata (21.1 μm) induced rosette-like structures with upregulation of EndMT and senescence markers, and large guttata (31.8 μm) caused the same rosette pattern with increased TUNEL-positive cells [60]. Compared to normal DM, cells surrounding large guttata displayed upregulated EndMT and cell senescence markers. These results highlight the importance of the guttata size in FECD. However, several known FECD intracellular markers were not found in this model, such as TCF4, Nrf2. Hence, the microenvironment may not be the only factor influencing FECD pathogenesis in vivo. In addition, the immortalized cell line used may not be the most accurate representation of in vivo FECD, since HCEnC-21T is a healthy HCEnC line known to have good proliferative ability [60,110].

Goyer et al., on the other hand, utilized CEnCs from end-stage FECD patients and seeded the cells onto decellularized DM of normal cornea to construct a corneal endothelium model. The engineered FECD tissue shows a significantly higher presence of fibronectin compared to the tissues seeded with healthy cells, implying that fibronectin may be involved in the early pathogenesis of the disease prior to the buildup of laminin and type IV collagen on DM [140].

#### 4.2.2. Animal Ex Vivo Corneal Tissue

Due to the limited availability of human corneal tissues, cornea specimens from other animal species, such as porcine corneal tissue and mouse ex vivo tissue, are considered potential alternatives for FECD studies, as they are more readily available [88,136,143] since they offer great potential for early-stage preliminary studies before being extended to human ex vivo corneal tissues [38,88].

Porcine corneas display endothelial cell death rates comparable to those of HCEnCs. A thinner corneal button can be created by removing the epithelial layer and part of the stromal layer [136]. This method does not require additional de-swelling compounds in the culture media, which is a desirable trait [136]. Conversely, an in vitro porcine model can also be prepared in organotypic culture, avoiding EndMT that may occur in human CEnC culture [136,147].

Rabiee et al. utilized an ex vivo porcine corneal tissue model to study oxidative stress and secretome in FECD. The researchers treated corneal tissues with varying concentrations of H_2_O_2_ and assessed their viability thereafter. The results of vital staining revealed that H_2_O_2_ treated porcine cornea tissues, without the secretome treatment (murine corneal mesenchymal stem cell secretome), suffered significant damage and exhibited opacity due to oxidative stress. This model is considered a reproducible representation of the effects of oxidative stress on the corneal endothelium [143].

Mouse ex vivo cornea tissue has also been employed for oxidative stress studies [88]. Jurkunas et al. performed a simple experiment in which ex vivo mouse corneal tissues were treated with varying concentrations of H_2_O_2_, and cell morphology and viability were evaluated. The results highlighted that oxidative stress led to cell morphological changes and apoptosis in both early- and late-onset forms, which mirror a potential FECD pathogenic mechanism [88].

### 4.3. In Vivo Animal Models

In vivo animal models are crucial for preclinical testing of drugs, treatments, and cell transplantation, as well as for investigating the pathogenesis of FECD. However, CEnC proliferation exhibits significant species-to-species variations. For example, rabbits, bovines, and murine models have higher proliferation rates during CEnC recovery, while non-human primates, felines, and porcine have limited proliferation rates, similar to humans [11,13,18,23,46,136,148]. Rabbit and feline models are typically used to study the functionality of primary corneal endothelial cells in corneal DM rather than to induce FECD, as their corneal size and thickness are similar to those of humans. Meanwhile, genetic mutations and UVA irradiation can be introduced into murine models to induce FECD phenotypes. Understanding the limitations of each animal model is important for designing and interpreting experiments in FECD research. Readers should refer to a recent review by Park et al. for an extensive review of various animal models of corneal endothelial dysfunction [149]. This section summarizes the utilization of different in vivo FECD models grouped according to species (Table 4).

#### 4.3.1. Murine Models

Murine (mouse) models are one of the most commonly used models in the FECD study due to the established model to introduce oxidative stress and robust techniques in the generation of genetically modified mice.

Two clinical subtypes of FECD (in Section 1 and Section 3) are early-onset, which is associated with Q455K and L450W mutations in the *COL8A2* gene [11], and the late-onset subtypes are associated with *TCF4*, *TCF8*, *SLC4A11*, and *COL8A2* genes [149,152].

A genetically engineered mouse model has been developed to study the late-onset form of FECD. Point mutations in the *COL8A2* gene have been associated with early-onset FECD [63,66]. In 2012, Jun et al. established homozygous WT and homozygous Col8a2*^Q455K/Q455K^* knock-in mutant mice to examine the features of FECD in humans [11]. The knock-in mice displayed essential elements extraordinarily similar to the prime characteristics of human FECD, including the pathogenesis progression in endothelial cell morphology. Pathologies like cell loss and guttata formation have also been observed in the knock-in model. This study demonstrated that Q455K substitutions in the *COL8A2* gene were sufficient to cause FECD in a mouse model. Q455K was correlated with DM thickening in young patients, but this was absent in the mouse Q455K model. It is worth noticing that the *COL8A2* gene differs between humans and mice, with only 94% matching amino acid identity; the equivalent 455 position was 451 in the mouse protein. Additionally, these studies used ten-month-old mice with CEnC densities of approximately 1240 cells/mm^2^. In contrast, severe phenotypes in human patients reach as low as 500–600 cells/mm^2^ [11], while healthy humans have approximately 3000 cells/mm^2^ [158]. Nonetheless, this study provides insight into the pathogenesis of FECD, as it is impossible to study the early pathophysiology using human tissue.

Another early onset murine model was generated with the L450W knock-in mutation in the *COL8A2* gene [68,152]. The L450W mutation presents a milder FECD phenotype than the Q455K mutation, with higher cell density, lower guttata density [68], and higher cell hexagonality percentage [152]. Interestingly, both mutations showed lower DM stiffness than the wild-type control DM from five-month-old mice. Alterations in DM stiffness induced changes in ECM and DM tissue compliance, leading to cell morphology and phenotype changes [68]. Additionally, mutations in the *COL8A2* gene in the early-onset form of FECD models caused endoplasmic reticulum (ER) stress and unfolded protein response activation, which induces further cell apoptosis [11,152].

The *TCF4* gene encodes the transcriptional factor E2-2, while *TCF8* encodes the transcriptional factor ZEB1 [159]. Han et al. used an *SLC4A11* knockout mouse model generated by gene deletion as a model for hereditary endothelial dystrophy [153]. Progressive corneal edema, increased CEnC size, decreased CEnC density, thickening of DM, and disorganization of collagen fibrils were observed with increasing age. Liu et al. developed a mutant ZEB1 knockout and a heterozygous mouse model for posterior corneal dystrophy [160]. Embryos and adult ZEB1 heterozygous mice exhibited abnormal corneal endothelial and keratocyte proliferation, corneal thickening, and corneolenticular and iridocorneal adhesions. The symptoms observed in ZEB1 null mice were more representative of posterior polymorphous corneal dystrophy (PPCD), where the expression of epithelial genes was observed in the endothelium, suggesting that ZEB1 may play a role in the suppression of an epithelial phenotype. Another study also reported a PPCD mouse model, which was a spontaneous mutant that arose in a mouse colony and exhibited metaplasia of the corneal endothelium [161]. Phenotype and gene expression were characterized, and ZEB1-related pathway genes were downregulated in the PPCD model. It is worth noticing that the ZEB1 and E2-2 promote EndMT and downregulation of E-Cadherin, which leads to the loss of cell-cell contact; therefore, ZEB1 would be a vital regulator of the EndMT process in CEnCs, which may have implications for the development and progression of FECD [59].

Recently, a double-mutant mouse model was created, which showed the presence of guttata, increased corneal thickness, decreased CEC density, and elevated ROS, with stromal lactate concentration indicating insufficient pump function [154]. Murugan et al. generated this double mutant model with a tamoxifen-inducible knockdown of *SLC4A11* and the *Q455K* knock-in mutation in *COL8A2*. This model shows important typical FECD phenotypes and has the potential for further study of FECD and its progression.

In vivo FECD models can also be created by exposure to oxidative stress. Liu et al. developed an FECD animal model by ultraviolet A (UVA) irradiation using C57BL/6 WT mice to explore the mechanisms of environmental and sex-dependent factors in FECD, as well as the reproduction of morphological and molecular changes of FECD [150]. They utilized mouse CE responsiveness to oxidative stress by UVA induction, which causes DNA damage and FECD. To establish a late-onset FECD mouse model, the eyes of mice were irradiated with UVA (250, 500, 750, and 1000 J/cm^2^). The hexagonal shape of mouse CEnCs was progressively lost, CEnC density decreased, and corneal thickness increased, which is consistent with the findings in human CE. Increased extracellular H_2_O_2_ levels were observed in the aqueous humor of both male and female mice. Notably, female mice were more susceptible to UVA. The sex-dependent effect of UVA on cytochrome P450 1B1 (CYP1B1), an enzyme that converts estrogen into DNA-damaging metabolites, in mouse corneas was observed in female but not in male mice. In another study, C57BL/6 WT mice (male and female, 7 to 15 weeks old) were used to investigate the link between CEnC senescence and EndMT with UVA light stress [151]. UVA (500J/cm^2^) irradiation induced EndMT and senescence in CEnCs. UVA irradiation also caused G2/M phase cell cycle re-entry in post-mitotic CEnCs in vivo, leading to senescence and fibrosis. The arrested senescent CEnCs then exhibited higher expression of EndMT genes, resulting in a profibrotic morphology. These findings indicate a clear link between the cell cycle status and EndMT.

Genetically modified mouse models can also be a useful tool for validating mechanistic studies in vivo. Ashraf et al. recently studied acute treatment with MN as a chemical stressor, UVA as a physiological stressor, and catechol estrogen on immortalized cell lines and ATM-knockout mice [129]. (see Section 4.1.2) UV irradiation was applied to ATM-wide-type and ATM knockout mice to validate that ATM activation is essential for UVA-induced cell cycle re-entry and senescence in vivo.

The advantages of using murine FECD models are their simple methodologies and straightforward techniques. In particular, UVA-induced FECD, post-irradiation morphological imaging, and cell density measurements are all practically achievable. Also, UVA-irradiated murine models are helpful in elucidating apoptosis mechanisms related to cell loss caused by oxidative stress. However, the genetic heterogeneity of FECD and the genetic differences between humans and mice are limitations.

#### 4.3.2. Rabbit Models

Rabbit models are often used to demonstrate the functionality of primary corneal endothelial cells in FECD research [13,21,24]. Rabbit CEnCs are different from human CEnCs, such as a higher proliferation rate and the ability to regenerate in wounds [148], but the rabbit model is still helpful in exploring therapeutics for corneal wound healing in a shorter time frame [13,24]. An example of using this advantageous proliferation is with rabbit CEnCs undergoing corneal wound healing, enhanced by the ROCK inhibitor Y-27632. The experiment was conducted by mechanically scraping the corneal endothelium in vivo, and the cornea was subsequently evaluated after 14 days [24]. Meekins et al. mechanically injured rabbit corneas and observed the progress of wound healing under the continous topical application of H-1152, another type of ROCK inhibitor. The experiment was evaluated within ten days [138]. H-1152 displayed a superior stimulatory effect compared to Y-27632 on CEnC migration and proliferation in vitro, suggesting that the topical administration of H-1152 enhances the healing of the injured corneal endothelium in vivo.

Rabbit models can also be used to test the efficacy of cell therapy. For instance, rabbit models have been used to assess cell injection therapy [162]. Okumura et al. showed that CEnCs regenerated in the rabbit corneal stroma, both with and without DM, after removing the CE. In cases where a 4-mm diameter DM in the optical zone was removed, cells repopulated the stromal surface, and after 14 days, no significant difference in CE was observed between the two groups. However, the recovery rates of the central corneal thickness and corneal transparency were slower in areas without DM or intact peripheral DM. It is also worth mentioning that after 14 days of cell injection, the cell density of the CE and thickness of the central CE remained the same in both areas, with or without DM. This study showed that removing diseased DM using a minor descemetorhexis procedure could improve the overall effectiveness of cell injection therapy. Rabbit models have also been used to evaluate and develop cell therapy protocols, such as prone position time for cell therapy [157], different cell sources [156], and cell tracking of transplanted cells [155].

Wound healing studies are commonly performed on in vivo rabbit models to recapitulate the wound healing process in humans. Apart from the proliferative difference, the mechanical strength of DM [163] and CEnC EndMT potentials [164] also differ between rabbits and humans. Nonetheless, in vivo endothelial scraping and other related wound-healing studies have revealed that healthy DM is critical for CEnC migration and proliferation [13], and how to assess cell-based therapy [162].

#### 4.3.3. Feline Models

Feline models are valuable for in vivo FECD research because of their similarity to the human corneal endothelium, which is non-proliferative in vivo. Similar to human CEnCs, feline CEnCs compensate for denuded areas through cell enlargement and migration [18,148]. The cell density and corneal thickness were also comparable to those of human corneas. The feline corneal diameter is 15.5–18 mm, similar to that of full-size human grafts, and thus can be handled using the same tools [18,148].

The feline model was also the first in vivo model studied with the transplantation of untransformed human FECD CEnCs as xenografts, to the best of researchers’ knowledge [18]. Haydari et al. seeded primary human FECD CEnCs onto a decellularized stromal carrier and cultured them for 8–15 days before transplanting them into feline eyes. The corneal endothelium pump function and transparency were restored seven days after transplantation. The recovery rate of FECD cells in feline eyes was higher than that in human patients’ corneas despite slight to moderate symptoms of FECD in transplanted corneas in the subsequent seven days. However, the recovery of the FECD endothelium was only partial. The transplanted FECD specimens remained thicker; more fibrillar substance buildup and decreased cell density were also observed in the “FECD model” [18]. This study provided a proof-of-concept “living” in vivo FECD model, which could potentially aid the development of novel therapies using human FECD cells in vivo. This model can also be used to observe FECD cell dysfunction (in the absence of the FECD DM microenvironment), molecular pathophysiology, and apoptosis. Lastly, this research provided the possibility of autografts for patients with FECD, as the function of FECD CEnCs was partially recovered in the feline model.

The feline model is also useful for the preclinical evaluation of CEnC reconstitution via intracameral CEnC injection. For example, allogeneic CEnCs were injected into the anterior chamber of a feline using a ROCK inhibitor [16]. The new endothelium in corneas grafted with 2 × 10^5^ CEnCs presented better functionality and anatomical integrity than that of corneas grafted with a density of 1 million CEnCs; however, it was still inferior to that of the normal controls. In contrast to other cell injection treatment studies in rabbit models [155,156,157,162], the allogenic injection treatment in this feline model could reconstitute an insufficiently functional corneal endothelium. Therefore, the authors questioned the effectiveness of cell injection therapy as a treatment for the incompletely functional endothelium, while the ROCK inhibitor alone may already be sufficient by stimulating the wound healing mechanism of the host peripheral cells. With a non-proliferative CEnC phenotype similar to that of humans, the feline model could be an excellent candidate for a preclinical animal model for cell therapy.

#### 4.3.4. Primate Models

The most human physiologically relevant model is the in vivo cynomolgus monkey CEnC model because of its extremely limited proliferation potential, similar to that of HCEnCs [23]. Okamura et al. conducted several studies on the ROCK inhibitor Y-27632 eye drop treatment that was introduced topically and evaluated for functionality in a non-human primate model with wounds created by transcorneal freezing [21,23]. The results demonstrated the in vivo therapeutic potential of ROCK inhibitorsfor treating FECD. Later studies have also confirmed the therapeutic potential of ROCK inhibitorsin clinical studies involving patients with FECD and corneal edema [21,23].

In summary, cellular injury in various in vivo animal models is typically introduced by mechanical or cryogenic damage or exposure to UV irradiation to induce oxidative stress. These models can recapitulate the CEnC damage response, proliferation, migration, and healing processes. However, the formation of guttata has not yet been well established in large animal models. In addition, the results and conclusions may not directly resemble human FECD pathology, especially when cell-guttata interactions in human FECD are also considered crucial components of FECD pathology. Meanwhile, animal models (e.g., rabbit, feline, or non-human primate) are ideal for proving physiological function of endothelial cells and evaluating the safety concerns of therapeutic treatment of FECD. Animal models can also be used to assess cell sheet transplantation or cell injection, especially after central descemetorhexis.

## 5. Conclusions

The pathologies of FECD remain to be elucidated even though significant progress has been made over the last decade. Various pathologies have been proposed with supporting evidence, such as mutations in the genes *COL8A2*, *SLC4A11*, and *TCF4*, as well as molecular mechanisms involving EndMT, oxidative stress from UV radiation and oxidants, and the presence of a guttata microenvironment. Studying these various mechanisms will advance our existing FECD models.

Furthermore, expanded primary human FECD CEnCs have been known to “restore” part of the healthy phenotype when removed from a guttata and cultured in vitro on a tissue culture plate. Thus, if researchers would like to investigate FECD pathogenesis, FECD cells may need to be observed and analyzed in a particular FECD model. A multitude of animal species have been used to model FECD, but none fully resemble the diseased phenotype and FECD microenvironment. Therefore, the biomimicking synthetic guttata FECD model may hold promise for modeling the microenvironment in vitro with a relatively simple setup, a feature analogous to the FECD phenotype. The lack of an FECD model hinders the development of alternative drug therapies, as the development of current drug therapies relies on in vitro cell culture models. To facilitate future research, improvements to the models are imperative not only to discover a unified pathology of FECD but also to facilitate novel drug development.

Overall, advancing FECD research and developing therapies for FECD require a multidisciplinary approach that combines the study of FECD pathology (including gene expression and induction by the external environment) and the development of FECD disease models. The successful establishment of FECD in vitro and in vivo models that closely recapitulate the FECD microenvironment is important for uncovering disease mechanisms and testing potential therapies. While FECD has been speculated to be caused by the combinatorial effects of genetic mutations and exogenous factors, studies using preclinical models with a combination of multiple stimuli, such as UVA combined with oxidative stress, genetic mutations, or guttata structure, are still limited. However, recently, an increasing number of studies have started to investigate FECD using a combination of multiple stimuli to better mimic the FECD pathology [129,150]. In addition, further exploration of environmental factors, such as oxidative stress, may reveal preventive approaches. Further research, especially in gene therapy and regenerative medicine, is important for overcoming the knowledge gap between FECD models and in vivo FECD etiology.

For additional insights into corneal physiology, readers can refer to the systematic review conducted by DelMonte et al. [50], and for a more rounded understanding of FECD pathology and pathogenesis, excellent reviews are available from Zhang et al. [165] and Ong Tone et al. [2].

## Figures and Tables

**Table 2:** Summary of the characteristics of various in vitro FECD models.

Model Species	Prime Characteristic	Pathological and Physiological Recapitulation	Pros	Cons	Ref.
**Primary HCEnCs**	In vitro expansion of primary healthy and FECD cells	Cell genetic mutation, molecular changes in cells	Closest recapitulation of the HCEnCs in vivo	Limited proliferative capacity. Primary FECD CEnCs isolated from old donors are challenging to expand	[16,41,97,98,99,100,101,102,103]
**Immortalized HCEnCs**	Viral oncogenes, Cellular Oncogene, RNA Interference, CRISPR/dCas9 immortalized FECD, and healthy cells	Cell genetic mutation, molecular changes in cells	Induced proliferative ability with active ion transport and full confluence. Less batch variability compared to primary cells	Less accurate biological information, specific to donors’ genetic profiles. Different from in vivo environment.	[44,45,48,59,88,93,103,104,105,106,107,108,109,110,111,112,113,114]
**iPSC-Derived CEnCs**	Cells derived from disease-specific iPSCs	Recapitulate cellular and molecular changes	Suitable for evaluating the precise mechanisms of cell death	Residual undifferentiated iPSCs present, limited reproducibility.	[115,116]
**Bovine corneal endothelial cells**	Cells harvested for in vitro drug testing	Recapitulate cellular and molecular changes	Ease of in vitro maintenance	Xenogeneic cells, do not fully recapitulate HCEnCs	[46]
**Primary HCEnCs on biomimicking synthetic guttata**	Hot embossed topographical patterns	Recapitulate the impaired DM and CEnCs microenvironment in vitro by mimicking guttata structure	Ability to control guttata size, morphology, stiffness, and spacing. Surfaces can be replicated, not reliant upon animal donors.	Not a complete representation of the in vivo environment.	[48,117]

**Table 3:** Summary of the characteristics of various ex vivo FECD model species.

Model and Species	Prime Characteristic	Pathological and Physiological Recapitulation	Pros	Cons	Ref.
**FECD ex vivo corneal tissue with cells (human)**	DM and CEnCs isolated from Stroma	FECD microenvironment, ECM	A close representation of the FECD in vivo, cells reside in native tissue	Not reusable, limited cell amount	[37,38,82,88,114,139]
**Normal ex vivo corneal tissue with cells (human)**	DM and CEnCs isolated from Stroma	Introducing oxidative stress via chemical or physical approach	A close representation of the normal environment in vivo	Not reusable	[113]
**FECD ex vivo corneal tissue with seeded immortalized cells (human)**	Decellularized tissue seeded with HCEnC-21T	FECD microenvironment, ECM	A close representation of the FECD in vivo. Studies using FECD DM with healthy cells are possible	HCEnC-21T may not be the closest representation of the diseased cell state; ex vivo tissue is not reusable	[60,140,141,142]
**Thin porcine corneal buttons**	Harvested as ex vivo tissues	Recapitulating human ex vivo corneal tissue	Cell death rates are comparable to HCEnCs. It could be used as organotypic culture ex vivo	Samples may be damaged when placed facing down	[60,113,136,143]
**Mouse corneal tissue**	Whole mount corneas, with CEnCs	Recapitulating human ex vivo corneal tissue	Great potential for early-stage preliminary studies	Different in physiology and limited representation of human FECD phenotype	[11]

**Table 4:** Summary of the characteristics of various in vivo FECD model species.

Model Species	Prime Characteristic	Pathological and Physiological Recapitulation	Pros	Cons	Ref.
**Murine (mouse)**	Exposure to ultraviolet A light	Recapitulate cellular DNA damage induced by UVA irradiation	Model for oxidative damage through ROS, in vivo recapitulation of the diseased environment	UVA exposure is considered the sole environmental factor in FECD induction, while many genes are involved in FECD development	[150,151]
	Mutation in *COL8A2* gene (including Q455K and L450W)	Early onset form of FECD	Model for early-onset FECD	Not representative of late-onset FECD	[11,63,66,68,152]
	SLC4A11 knockout mouse	Late-onset form of FECD and congenital hereditary endothelia dystrophy	Progressive corneal edema, vacuolated CEnCs, thickened DM with age.	Phenotypic changes are possibly milder than those in human patients	[11,63,66,68,152,153]
	Double mutation: *SLC4A11* knockdown and *Q455k* knock-in	Presence of guttata, increased corneal thickness, decreased CEC density, elevated ROS	Model shows important typical FECD phenotypes	Not correspond to a specific genotype associated with FECD in humans.	[154]
**Rabbit**	Mechanical or cryogenic injury	Microenvironment changes to study CEnC dysfunction and corneal edema	Migration and proliferation model for the regeneration of wounds. Helpful in exploring therapeutics in a shorter time frame.	Human CEnCs do not proliferate, unlike rabbit CEnCs.	[13,21,24,138,155,156,157]
**Feline**	Partial DM removal in vivo	Recapitulate impaired DM and CEnCs	Does not proliferate in vivo. Cell density and corneal thickness are similar to humans. Human xenograft transplantations into felines are well tolerated.	Not a full recapitulation of human DM and CEnCs in vivo	[16,18,148]
**Cynomolgus monkey**	Mechanical or cryogenic injury	Microenvironment changes to study CEnC dysfunction	Most similar and physiological relevant to human CEnCs	Not a full recapitulation of human FECD	[19,21,23]

## Data Availability

Data sharing is not applicable to this article, as no new data were created or analyzed in this study.

## Abbreviations

The following abbreviations are used in this manuscript:

FECD	Fuchs Endothelial Corneal Dystrophy
CEnCs	corneal endothelial cells
CE	corneal endothelium
ECM	extracellular matrix
COL8A2	collagen VIII α2-subunit chain
TCF4	transcription factor 4
DM	Descemet’s Membrane
ABL	anterior banded layer
PNBL	posterior non-banded layer
EC	endothelial cells
SLC4A11	Solute carrier family 4-member 11 bicarbonate transport protein
EndMT	endothelial-mesenchymal transition
TGF-β	transforming growth factor beta
ZEB1	E-box-binding homeobox 1
LOXHD1	lipoxygenase homology domains 1
AGBL1	ATP/GTP binding protein-like 1
ER	endoplasmic reticulum
UPR	unfolded protein response
CTG	cytosine–thymine–guanine
TNR	trinucleotide repeat
E2-2	E-protein family
MBNL1	Muscleblind-like 1 protein
mRNA	messenger RNA
CHED2	congenital hereditary endothelial corneal dystrophy type 2
ROS	reactive oxygen species
EMT	epithelial-to-mesenchymal transition
UVA	ultraviolet A
CDKN1a	cyclin-dependent kinase inhibitor 1A protein
PRDX2	Peroxiredoxin 2
tBHP	tert-Butyl hydroperoxide
SOD2	superoxide dismutase 2
HCEnCs	human CEnCs
mtDNA	mitochondrial DNA
NCDP	neural crest-derived progenitor
iPSC	induced pluripotent stem cells
HPV	Human papillomavirus
hTERT	human Telomerase Reverse Transcriptase
MN	menadione
NQO1	NAD(P)H quinone oxidoreductase 1
PDRN	polydeoxyribonucleotide
iFECDCs	Immortalized FECD cell lines
iHCEnCs	immortalization of CEnCs from healthy donors
Mfn2	Mitofusin 2
ATM	Ataxia-Telangiectasia Mutated
PI3K	phosphatidyl inositol 3-kinase
dCas9	CRISPR-dead Cas9
GelMA	gelatin methacrylate
BCE	bovine corneal endothelial
nDNA	nuclear DNA
Nrf2	nuclear factor erythroid 2-related factor 2
SFN	sulforaphane
GFP	green fluorescent protein
LNP	lipid nanoparticles
DSO	Descemet Stripping Only
PPCD	posterior polymorphous corneal dystrophy
CYP1B1	cytochrome P450 1B1
